# Enhanced antibacterial activity of porous chitosan-based hydrogels crosslinked with gelatin and metal ions

**DOI:** 10.1038/s41598-024-58174-9

**Published:** 2024-03-29

**Authors:** Bahareh Farasati Far, Mohammad Reza Naimi-Jamal, Mehdi Jahanbakhshi, Alireza Hadizadeh, Shiva Dehghan, Shiva Hadizadeh

**Affiliations:** 1 Research Laboratory of Green Organic Synthesis and Polymers, Department of Chemistry, Iran University of Science and Technology, Narmak, Tehran, Iran; 2 School of Chemical Engineering, College of Engineering, University of Tehran, Tehran, Iran; 3 Research Center for Advanced Technologies in Cardiovascular Medicine, Cardiovascular Diseases Research Institute, Tehran University of Medical Sciences, Tehran, Iran; 4 School of Pharmacy, Shiraz University of Medical Sciences, Shiraz, Iran; 5 Women Reproductive Health Research Center, Tabriz University of Medical Sciences, Tabriz, Iran

**Keywords:** Microbiology, Chemistry, Engineering

## Abstract

Addressing the increasing drug resistance in pathogenic microbes, a significant threat to public health, calls for the development of innovative antibacterial agents with versatile capabilities. To enhance the antimicrobial activity of non-toxic biomaterials in this regard, this study focuses on novel, cost-effective chitosan (CS)-based hydrogels, crosslinked using gelatin (GEL), formaldehyde, and metallic salts (Ag^+^, Cu^2+^, and Zn^2+^). These hydrogels are formed by mixing CS and GEL with formaldehyde, creating iminium ion crosslinks with metallic salts without hazardous crosslinkers. Characterization techniques like FTIR, XRD, FESEM, EDX, and rheological tests were employed. FTIR analysis showed metal ions binding to amino and hydroxyl groups on CS, enhancing hydrogelation. FESEM revealed that freeze-dried hydrogels possess a crosslinked, porous structure influenced by various metal ions. Antibacterial testing against gram-negative and gram-positive bacteria demonstrated significant bacterial growth inhibition. CS-based hydrogels containing metal ions showed reduced MIC and MBC values against *Staphylococcus aureus* (0.5, 8, 16 µg/mL) and *Escherichia coli* (1, 16, 8 µg/mL) for CS-g-GEL-Ag^+^, CS-g-GEL-Cu^2+^, and CS-g-GEL-Zn^2+^. MTT assay results confirmed high biocompatibility (84.27%, 85.24%, 84.96% viability at 10 µg/mL) for CS-based hydrogels towards HFF-1 cells over 48 h. Therefore, due to their non-toxic nature, these CS hydrogels are promising for antibacterial applications.

## Introduction

The transmission of infectious diseases caused by harmful bacteria continues to present a threat to public health^1^. World Health Organization data shows that infectious diseases are the world's second leading mortality^2^. It is also noteworthy that the misuse of conventional antibiotics has resulted in an escalation of drug resistance caused by pathogenic microbe^3,4^. Therefore, it is necessary to prepare efficient antibacterial agents and materials, especially those with multifunctional capabilities and ease of fabrication.

It has been found that hydrogels that exhibit good mechanical properties and possess antibacterial activity are suitable candidates for many biomedical applications in this regard^5^. With innovative approaches such as the incorporation of metallic ions and nanoparticles, these hydrogels offer a sophisticated platform to address the pressing global challenge of antimicrobial resistance. By understanding the intricate structure–function relationships at the nanoscale, researchers can engineer hydrogels with tailored antimicrobial properties, particularly crucial in biomedical applications like tissue engineering and drug delivery^6,7^.

Polymers, particularly chitosan (CS), are extensively employed in antimicrobial applications such as wound dressing due to their multifaceted advantages^8^. CS, with inherent antimicrobial properties derived from its cationic nature, interacts with bacterial cell membranes, disrupting them and preventing infection. Its biocompatibility ensures safety, and hemostatic capabilities aid in blood clotting^3,9^. The hydrophilic nature of polymers, including CS, supports moisture retention, fostering an optimal wound healing environment^10,11^. Further, these polymers can be tailored to sustainably release antimicrobial agents, promoting prolonged efficacy. CS also serves as a scaffold for tissue regeneration, contributing to wound closure. The flexibility and conformability of polymer-based dressings enhance patient comfort and optimize contact with the wound site^12^. Therefore, CS and similar polymers offer a versatile and effective solution for antimicrobial dressings, addressing key aspects of infection prevention^13^.

While the main crosslinkers for CS, including glutaraldehyde, genipin, tripolyphosphate (TPP), sodium tripolyphosphate (STPP), dibasic sodium phosphate, polyphosphates, ethylenediamine (EDA), tannic acid, and formaldehyde, serve specific purposes in enhancing the properties of chitosan-based materials, each comes with its own set of drawbacks^14^. Glutaraldehyde is associated with cytotoxicity and potential allergenicity, while genipin exhibits slow crosslinking kinetics and cost considerations^15^. TPP and STPP offer limited control over crosslinking density and may result in burst release in drug delivery applications^16^. Dibasic sodium phosphate, polyphosphates, and EDA have challenges related to control over crosslinking and potential cytotoxicity^17^. Tannic acid faces limitations in solubility and potential incomplete crosslinking^18^, while formaldehyde poses significant toxicity and safety concerns^19^. Researchers continuously explore alternative crosslinkers and optimization strategies to address these drawbacks and enhance the applicability of chitosan-based materials in diverse applications. The crosslinkers used in many commercial hydrogels are often costly^20^. Therefore, it is important to develop and use environmentally friendly and bio-based crosslinkers^21^.

In this study, gelatin (GEL) emerged as a favorable candidate for the development of the hydrogel. GEL is a biodegradable polypeptide that can serve as a crosslinker for polysaccharide-based hydrogels and is derived from collagen in animal tissues like porcine skin^22^. Its non-toxicity, biodegradability, and biocompatibility make it a promising crosslinker. GEL contains various functional groups, including amino (–NH_2_) and carboxyl (–COOH) groups, which act as sites for chemical reactions^23^. These reactive groups provide attachment points for other molecules or polymers, allowing GEL to form covalent bonds with them^24^. As a result, for this study, supramolecular hydrogels with antibacterial properties were prepared via GEL and the complexation of CS in the presence of Ag^+^, Cu^2+^, and Zn^2+^ transition metal ions. This approach could lead to the development of more effective antibacterial hydrogels with improved healing outcomes for patients.

Utilizing ions such as silver (Ag^+^), zinc (Zn^2+^), and copper (Cu^2+^) as alternatives to traditional crosslinkers in chitosan-based materials offers a multitude of advantages^25^. These metal ions bring intrinsic antimicrobial properties to the formulations, providing a natural defense against bacterial colonization, particularly valuable in applications like wound dressings and biomedical materials. Their biocompatibility at controlled concentrations ensures minimal toxicity concerns, aligning with the stringent requirements for biomedical applications^26^. The gradual release of these ions from the CS matrix facilitates controlled release mechanisms, a valuable feature in drug delivery systems^27^. Moreover, silver, zinc, and copper have demonstrated the ability to inhibit bacterial growth while enhance wound healing processes, promoting cell migration, proliferation, and tissue regeneration. Their broad-spectrum antimicrobial activity against bacteria, fungi, and viruses further reinforces their efficacy^28^.

Previous studies also reported some ions with other polymers. Klinkajon and Supaphol prepared Cu^2+^ crosslinked alginate hydrogels that have shown good antibacterial activity against both gram-negative and gram-positive bacteria. In addition, these hydrogels could coagulate fibrin in blood coagulation tests^29^. An alginate solution with Ca^2+^ is crosslinked to form hydrogels by ionic crosslinking for wound dressing applications^30^. The ionic crosslinking of alginate can also be achieved using divalent metal ions, including Mg^2+^, Fe^2+^, Ba^2+^, and Sr^2+^. Metal ions can also be incorporated into other biopolymers to create hydrogels^31^. It has been shown that CS can form hydrogels when combined with transition metals^32^. The use of transition metal ions in ultrafast multi-stimuli-responsive CS hydrogels has also been reported^33^.

In contrast to common chemical crosslinkers, the cytotoxicity of these metal ions is decreased, hence enhancing their biocompatibility. Moreover, their protection of the environment is consistent with the concepts of green chemistry. The integration of various metal ions or their synergistic application with other antimicrobial agents offers prospects for enhancing overall efficiency while reducing the potential for resistance emergence^34^. Ongoing research continues to optimize the incorporation of these metal ions into chitosan-based materials, harnessing their antimicrobial prowess for diverse applications in a safe and effective manner.

This study aims to elucidate the antibacterial efficacy and biocompatibility of the synthesized hydrogels, paving the way for potential biomedical applications. This study aims to introduce an antibacterial hydrogel network without toxic crosslinkers, possessing favorable physical properties and demonstrating effective biological performance, including synergistic antibacterial effects. hence, supramolecular hydrogels with antibacterial properties were prepared via GEL and the complexation of CS in the presence of Ag^+^, Cu^2+^, and Zn^2+^ transition metal ions. To obtain the hydrogels, GEL, and metal ions were added to CS solution under acidic pH. FTIR, XRD, FESEM, EDX, and rheological analyses were conducted on the hydrogels. Hydrogels were also evaluated for their antibacterial properties against *Escherichia coli* (gram-negative) and *Staphylococcus aureus* (gram-positive) using the agar well and disk diffusion methods. In vitro, MICs and MBCs were determined for the materials against the mentioned bacteria. To the best of our knowledge, it is the first study to describe the fabrication of antibacterial CS crosslinked with GEL and metal ions supramolecular hydrogels.

## Results and discussion

### Synthesis mechanism of chitosan-based hydrogels

**Figure 1:**
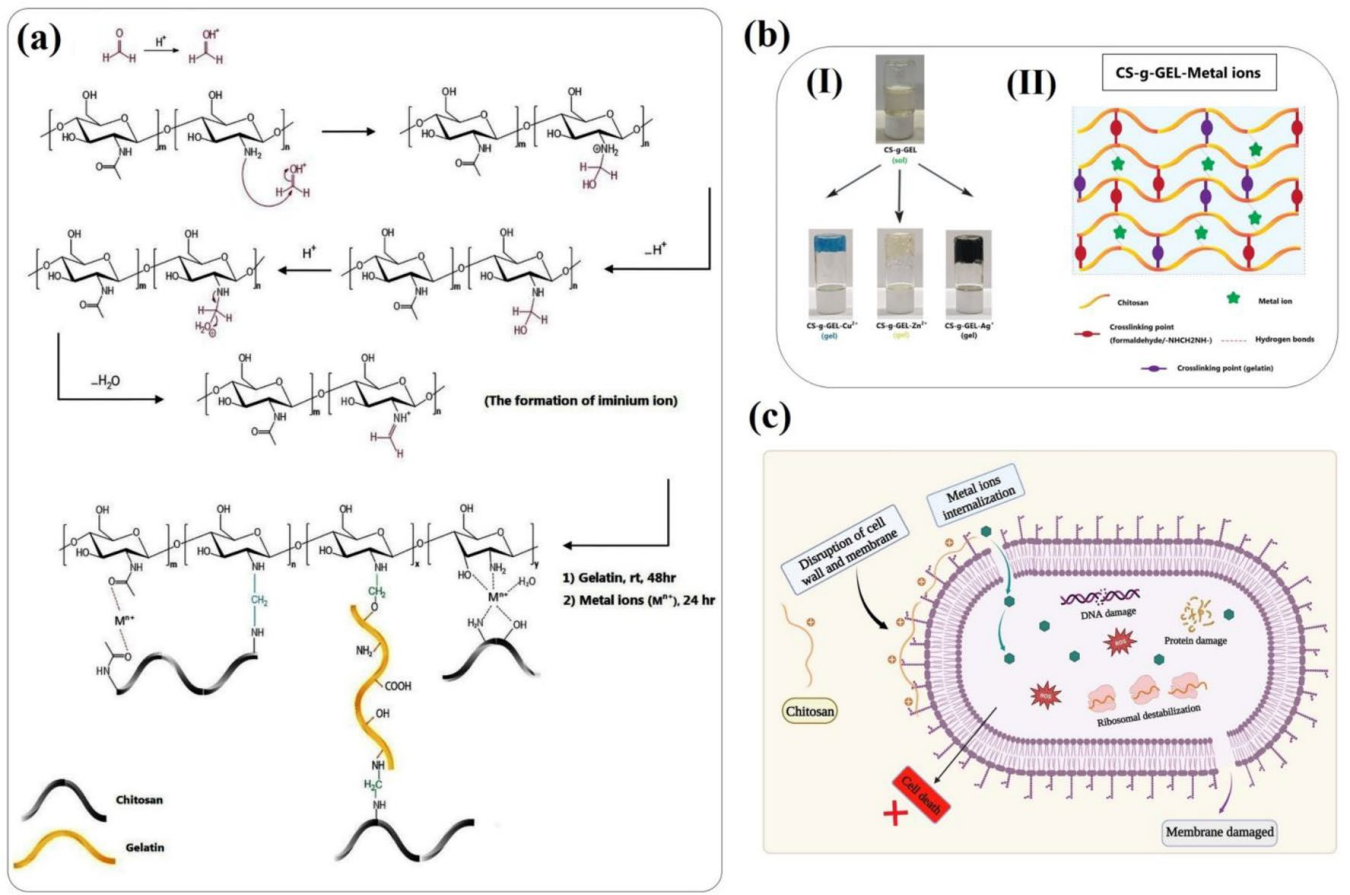
(**a**) Schematic of synthesis of CS-g-GEL-Metal ions, (**b**) photographs of reversed vial test method (I) and design strategy for CS-g-GEL-Metal ions hydrogels (II), and (**c**) antibacterial mechanism of CS-g-GEL-Metal ions hydrogels.

Hydrogels made from CS can be made using several formulations. In this study, covalent chemical bonds (covalent bonds between CS and formaldehyde, and formaldehyde with POM) and non-covalent physical interactions (hydrogen bonds between CS and ammonium-ion) have contributed to the formation of networks. As shown in Fig. 1a, b, gelatin attachment to CS is a result of physical interaction as well as a covalent attachment process. In acidic environments (NH_3_^+^), CS is protonated since it contains amine groups, providing its dissolution in water. In order to provide sufficient functional groups and establish a covalent bond between CS and GEL, formaldehyde was attached covalently to CS through interactions with amino groups (Fig. 1). When CS is chemically activated; amino groups bond with free aldehydes. Gelatin was then added to the CS solution as a crosslinking agent. The mixture of activated CS-g-GEL and metallic salt solutions produced the hydrogels within the gelation time reported in Table 1. A reversed vial test was used to visually identify hydrogel formation. In this study, the hydrogels were formed without the addition of NaOH, which was different from previous studies reported in the literature^35^. A stable, uniform, and elastic hydrogel was produced as a result of the mentioned method. According to Table 1, the gelation time for the CS-g-GEL-Ag^+^ hydrogel is 3 h, while the gelation time for CS-g-GEL-Cu^2+^ is 6 h, and CS-g-GEL-Zn^2+^ is 5 h at the same pH range. These values suggest that the CS-g-GEL-Ag^+^ hydrogel has the fastest gelation time, followed by the CS-g-GEL-Zn^2+^ hydrogel and then the CS-g-GEL-Cu^2+^ hydrogel. In addition, CS-g-GEL-Ag^+^ and CS-g-GEL-Cu^2+^ hydrogels showed stronger mechanical properties than CS-g-GEL-Zn^2+^ hydrogel, and their hydrogelation processes were faster. Additionally, we have observed several different phenomena when the ratio between the metal ions and CS-g-GEL ratio is increased or decreased, including partial gelation of hydrogels, a long period of hydrogelation, and a very weak hydrogel that can easily be converted into solution by shaking for an extended period of time. The specific effects observed when altering the ratio between the metal ions and the CS-g-GEL ratio depend on the nature of the metal ions and their interactions with CS. Increasing or decreasing the metal ion concentration can affect the balance between metal ion complexation and CS chain entanglement, leading to distinct gelation behaviors. The partial gelation observed could be attributed to incomplete crosslinking due to insufficient metal ion complexation with amino groups of CS, resulting in a gel with regions of lower mechanical strength and stability^36^. A prolonged period of hydrogelation may indicate a slower gelation rate caused by the need for more time to achieve sufficient crosslinking or the formation of a more intricate network structure. The formation of a weak hydrogel that can be easily converted into a solution by shaking for an extended period of time suggests a reversible gelation process, where the metal ion complexes can dissociate and reform, resulting in a gel-to-solution transition^37,38^. CS and metal ion complexes are known to exhibit antimicrobial activity against a wide range of bacterial species. The exact mechanism of action of these complexes is not fully understood, but it is thought to involve multiple steps (Fig. 1c). One proposed mechanism is that the positively charged CS-g-GEL-Metal ions hydrogels can bind to the negatively charged bacterial cell wall, disrupting its structure and causing the release of cellular contents. This can lead to cell death or inhibition of growth. Another proposed mechanism is that the metal ions in the complex can generate reactive oxygen species (ROS) that damage the bacterial cell membrane and cellular components, leading to cell death^39^. Additionally, CS-g-GEL-Metal ions complexes may interfere with bacterial DNA replication and transcription, leading to inhibition of growth and reproduction. Therefore, the antimicrobial activity of CS-g-GEL-Metal ions hydrogels likely involves a combination of these mechanisms, and the specific mechanism may depend on the type of metal ion used and the bacterial species being targeted^40^.

**Table 1:** The gelation time of various CS-g-GEL-Metal ions hydrogels.

Hydrogels	Studied pH range	Gelation time (h)
CS-g-GEL-Ag^+^	3.51–5.8	3
CS-g-GEL-Cu^2+^	3.51–5.8	6
CS-g-GEL-Zn^2+^	3.51–5.8	5

### Characterization of hydrogels

#### ^1^H-NMR structural analysis: grafting confirmation

**Figure 2:**
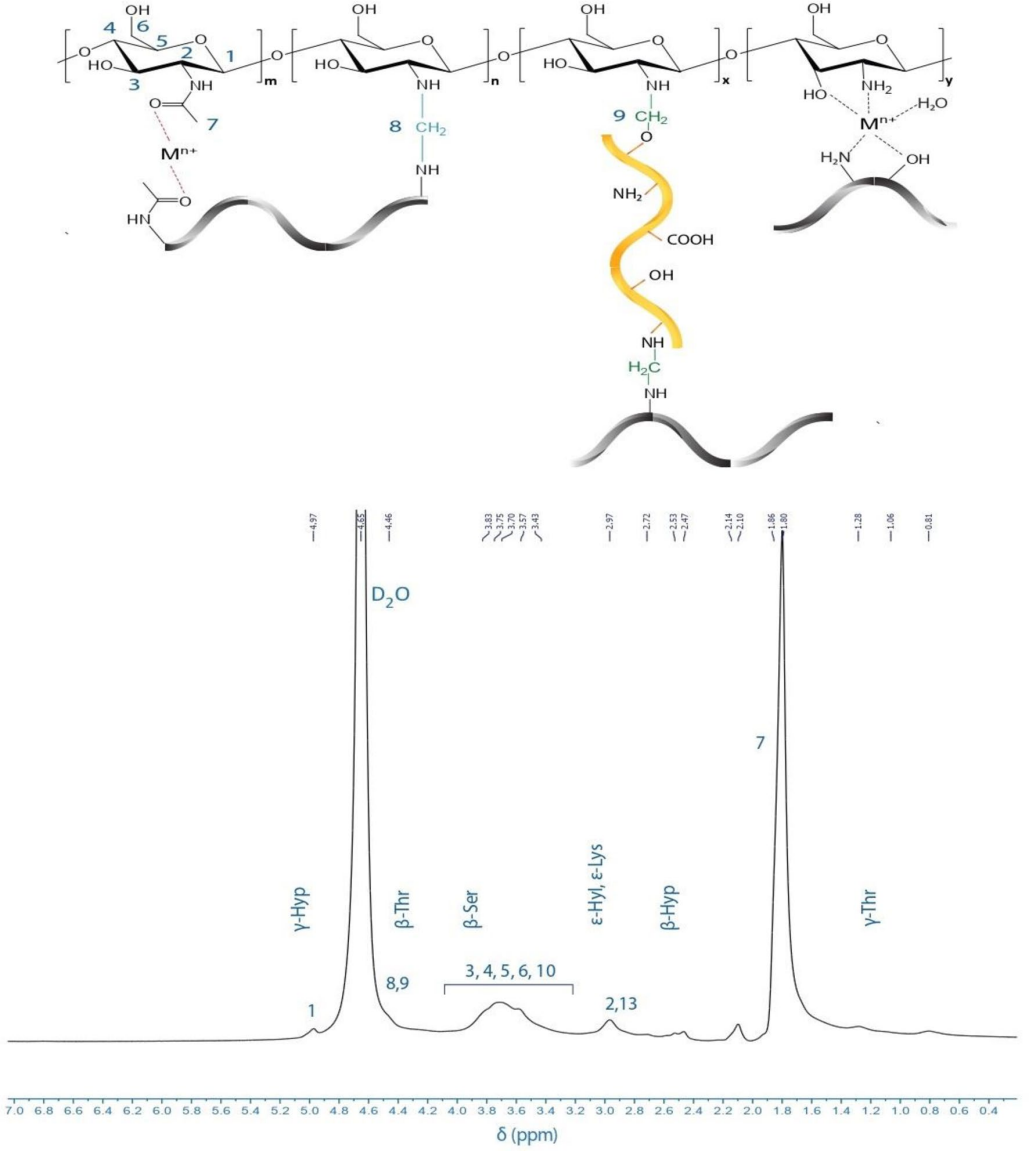
^1^HNMR spectrum of CS-g-GEL hydrogel (D_2_O, 25 °C)^22^.

^1^H NMR spectroscopy in D_2_O was conducted to investigate CS-g-GEL hydrogel. As shown in Fig. 2, the peak at 1.80 ppm can be assigned to the acetyl group, while the peak at 2.97 ppm corresponds to the H2 proton of CS. Also, the signals ranging from 3.43 and 4.00 ppm are attributed to the H3–H6 and protons of CS and the peak at 4.46 ppm can be assigned to methylene groups that are formed when GEL and CS are crosslinked (H8, H9). Farasati Far et al. identified that the resonance observed between 4.57 and 4.66 ppm is associated with the methylene groups resulting from the cross-linking between glycerol and chitosan^41^. Signals in the range of 4.97 ppm are caused by hydrogen bonds to anomeric carbons (H1). In Table 2 and Fig. 2, the result of the ^1^H NMR analysis of typical peaks of the amino acids in GEL has been identified (lysine, hydroxylysine, threonine, serine, and hydroxyproline). When GEL was grafted into CS, the characteristic peaks associated with the amino acids shifted, proving the grafting process^42^. As an example, the peak at 1.28 ppm can be associated with γ-threonine (CH_3_). Table 2 provide characteristic proton of the amino acids of gelatin involved in CS-g-GEL. The observed peaks corresponding to various functional groups and amino acids confirm the successful grafting of GEL into CS and provide evidence of the crosslinking process. The presence of observed peaks corresponding to different functional groups provides strong evidence supporting the successful grafting of GEL into CS. These peaks confirm the occurrence of the grafting process and validate its effectiveness.

**Table 2:** Characteristic proton of the amino acids of Gelatin involved in CS-g-GEL.

Characteristic proton of the amino acids of Gelatin involved in CS-g-GEL	Amino acid abbreviation	Chemical shift of protons (ppm)
γ-threonine (CH_3_)	γ-Thr	1.28
β-threonine (CH)	β-Thr	4.46
β-serine (CH_2_)	β-Ser	3.83
ε-hydroxylysine (CH_2_)	ε-Hyl	2.97
ε-lysine (CH_2_)	ε-Lys	2.97
γ-hydroxyproline (CH)	γ-Hyp	4.97
β-hydroxyproline (CH_2_)	β-Hyp	2.47

#### FT-IR analysis: elucidating molecular structure

**Figure 3:**
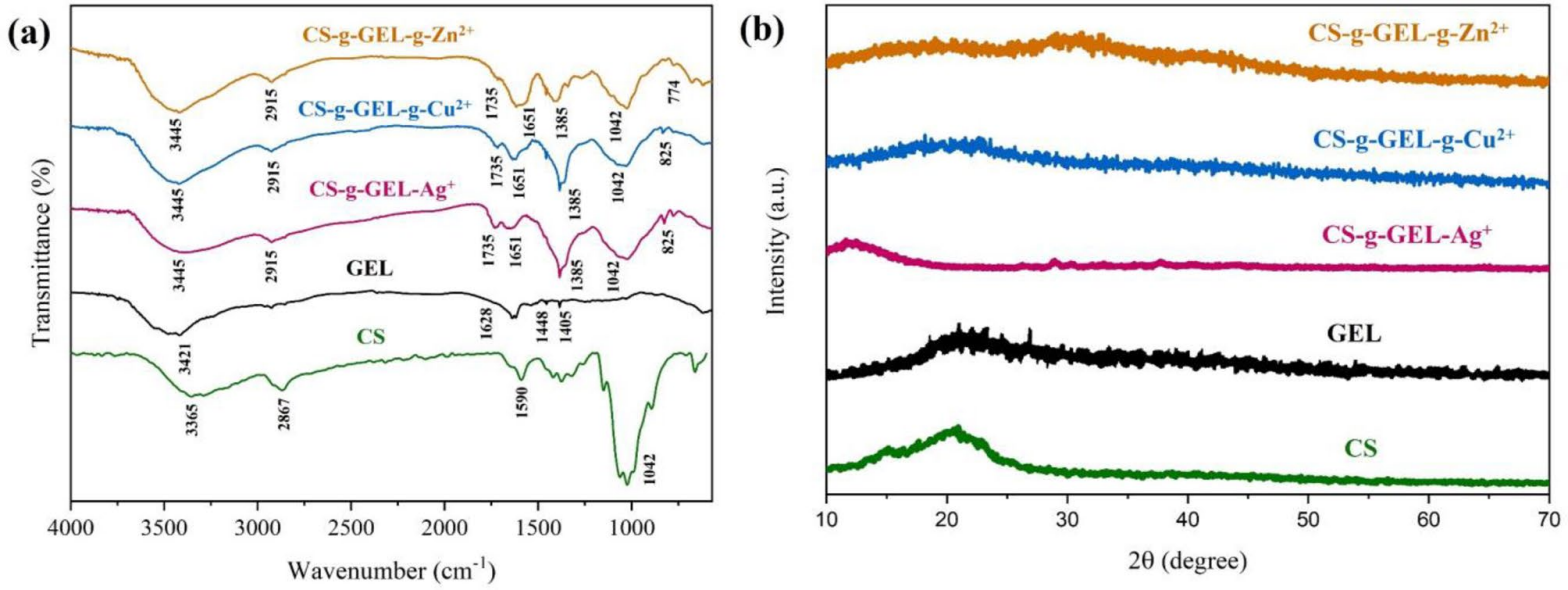
(**a**) FTIR spectra of CS, GEL, CS-g-GEL-Ag^+^, CS-g-GEL-Cu^2+^, and CS-g-GEL-Zn^2+^ hydrogels, and (**b**) XRD patterns of CS, GEL, CS-g-GEL-Ag^+^, CS-g-GEL-Cu^2+^, and CS-g-GEL-Zn^2+^ hydrogels.

Figure 3a shows the FTIR spectra of the chitosan, gelatin, and CS-g-GEL-Metal ions hydrogels. As shown in Fig. 3a, the broad peak at 3365 cm^−1^ observed in CS can be assigned to the stretching vibration of –OH. The reason for the absorption at 1590 cm^−1^ could also be assigned to the amide groups. There is a stretching frequency of 2867 cm^−1^ observed for the methylene group. A broad absorption band at 3300–3450 cm^−1^ was observed in all hydrogel spectra as a result of stretching vibrations of O–H and N–H. For gelatin, the peak at 1628 cm^−1^ is associated with C=O (amide I), while the peaks at 1405 and 1448 cm^−1^ are assigned to the N–H deformation vibration. A broad absorption band at 3421 cm^−1^ is a result of stretching vibrations of O–H and N–H. In CS-g-GEL-Metal ion hydrogels, OH and NH bands shift to higher wavenumbers because amino and hydroxyl groups are involved in complexation, which may suggest stronger intermolecular interactions through hydrogen bonds. This suggests chitosan contains lone pairs of oxygen electrons that interact with metal ions to produce this peak^35^. The peak at 1385 cm^−1^ is due to the coupling of (–C–N–) stretching vibration. The absorption band at 1042 cm^−1^ is assigned to the stretching of the (–C–O–) bond. According to Fig. 3a, a gradual increase in the intensity of the peaks at 1651 cm^−1^ and new peak at 1735 cm^−1^ in CS-g-GEL-Metal ions are suggestive of an increase in Ag^+^-NH_2_, Cu^2+^-NH_2_, and Zn^2+^-NH_2_ and interaction which could suggest the presence of carbonyl groups in the form of esters or ketones within the molecular structure of the hydrogel^35^. It was found that the absorption peaks at 825 cm^−1^ in the CS-g-GEL-Ag^+^ CS-g-GEL-Cu^2+^ hydrogel spectrum and in the CS-g-GEL-Zn^2+^ hydrogel spectrum at 774 cm^−1^ were caused by the coordination of water molecules^43^. Additionally, the bands observed in CS-g-GEL-Metal ions hydrogels at 730–825 cm^−1^ could be attributed to metal–oxygen bonds (MOs)^44^. As a result, metal ions had a complexation with CS as a result of OH and NH_2_ groups.

#### XRD analysis

XRD patterns of chitosan (a), gelatin (b), CS-g-GEL-Ag^+^ (c), CS-g-GEL-Cu^2+^ (d), and CS-g-GEL-Zn^2+^ (e) hydrogels in the 2*θ* range of 10–70 (°) is shown in Fig. 3b. Chitosan's crystalline character is attributed to the strong intramolecular hydrogen bond that distinguishes it from most carbohydrates. Pure chitosan and gelatin powder exhibited an intense peak approximately at 2θ = 20.96°, while the CS-g-GEL-Metal ions hydrogels displayed a broader and less intense peak in the same degree for CS-g-GEL-Ag^+^ (c), CS-g-GEL-Cu^2+^ (d), and CS-g-GEL-Zn^2+^, respectively. In Fig. 3b**,** the broad peak at 2θ = 36.18° (002) and 28.98 (101) clearly shows the presence of Ag in CS-g-GEL-Ag^+^ hydrogel. For CS-g-GEL-Cu2^+^, the broad peak at 2θ = 19.68° (111) indicates the presence of copper. In Fig. 3b, the peak at 2θ = 36.49° (111) and 44.02 (210) clearly shows the presence of zinc in CS-g-GEL-Zn^2+^ hydrogel. There was a change in XRD patterns, which could be attributed to the formation of complexes between CS-g-GEL and metal ions through –OH and –NH_2_ binding sites, affecting the hydrogen bonding and destroying the crystal structure of the compound. In all three kinds of hydrogels, the XRD results revealed amorphous structures. Chitosan-Zn complexes^45^, chitosan-Ag^33^, carboxymethyl chitosan-Cu, carboxymethyl chitosan-Ag and carboxymethyl chitosan-Zn hydrogels have been reported in literature with similar XRD patterns^35^. In a study conducted by Wang et al., the XRD patterns of chitosan-metal complexes exhibited significant changes, with the original peaks of chitosan either disappearing or weakening. It was reported that the vanishing of these diffraction peaks is due to the chelation of metal ions with the –NH_2_ and –OH groups^46^.

### Rheological properties of hydrogels

**Figure 4:**
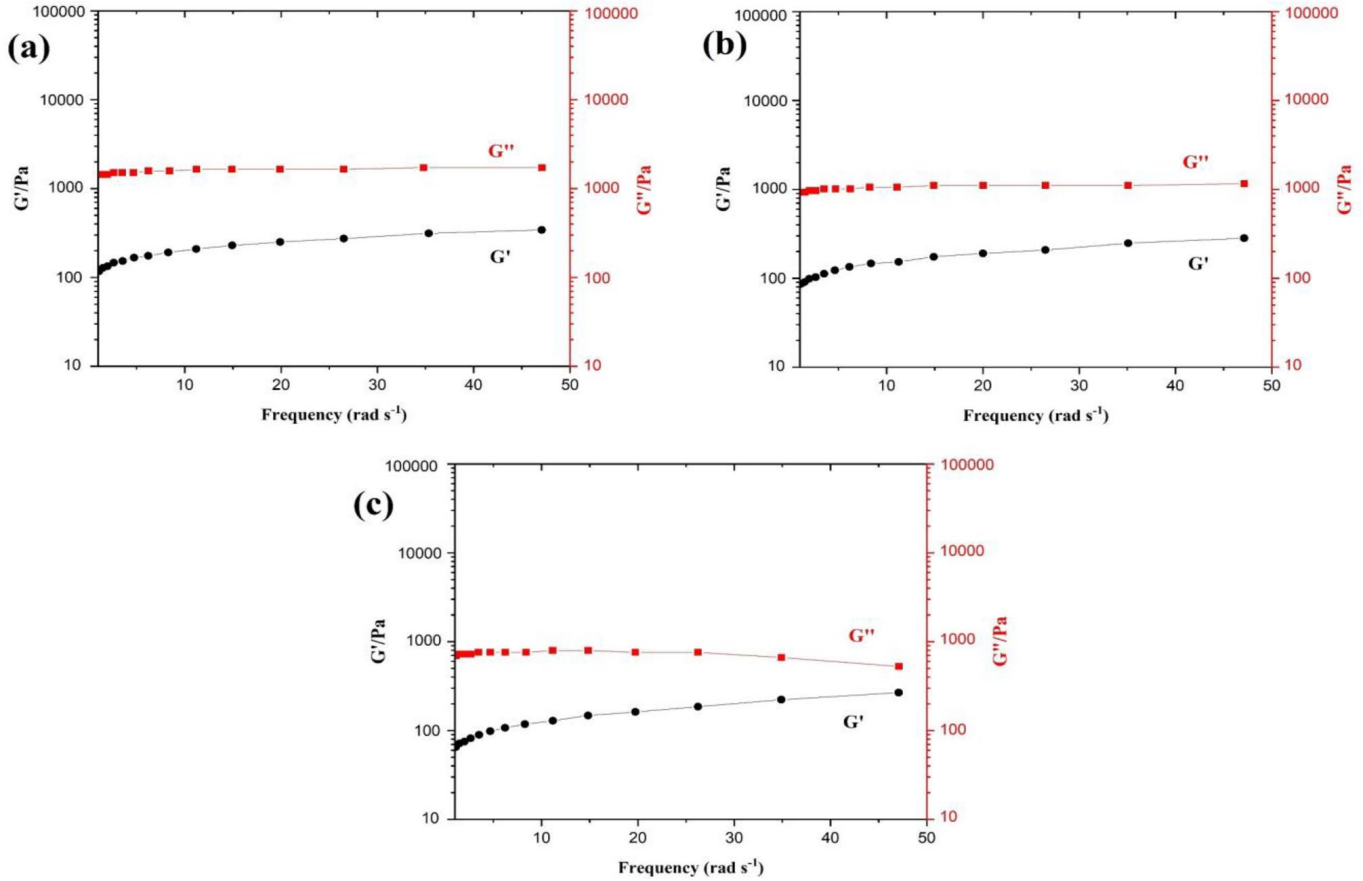
Frequency sweeps of the (**a**) CS-g-GEL-Ag^+^ (**b**), CS-g-GEL-Cu^2+^ (**c**), and CS-g-GEL-Zn^2+^ (**e**) hydrogels. (Storage (G′) and loss (G″) moduli).

Hydrogels are soft solids that exhibit viscoelastic properties, allowing them to store and dissipate energy^33^. In dynamic rheology, the viscoelastic characteristics of hydrogels are measured in terms of storage (G′) and loss (G″) moduli, which represent the stress in-phase and out-of-phase, respectively, in response to an imposed oscillatory deformation. The results showed that all three types of CS-g-GEL-Metal ions hydrogels had high mechanical strength (Fig. 4). The observation that the storage modulus (G′) was always greater than the loss modulus (G″) in all CS-g-GEL-Metal ions hydrogels, indicating their elastic nature as soft solids, is consistent with the behavior of other hydrogels reported in the literature. For example, Wahid et al. reported that carboxymethyl chitosan-Cu, carboxymethyl chitosan-Ag, and carboxymethyl chitosan-Zn hydrogels displayed high mechanical strength and elastic nature^35^. In a study by Chaudhuri et al., they examined the influence of substrate stiffness on cell spreading, focal adhesions, and cellular behavior. They investigated the impact of viscoelastic properties on tissue growth and development^47^.

When the storage modulus is greater than the loss modulus, the material is said to be in the "elastic" region, meaning that it can deform under stress and then return to its original shape when the stress is removed. This behavior is characteristic of soft solids such as hydrogels, which can undergo large deformations without breaking or losing their shape^35^. The elastic nature of the CS-g-GEL-Metal ions hydrogels is likely due to the crosslinked network structure of the hydrogel, which allows it to resist deformation and maintain its shape.

### FESEM morphological investigations

**Figure 5:**
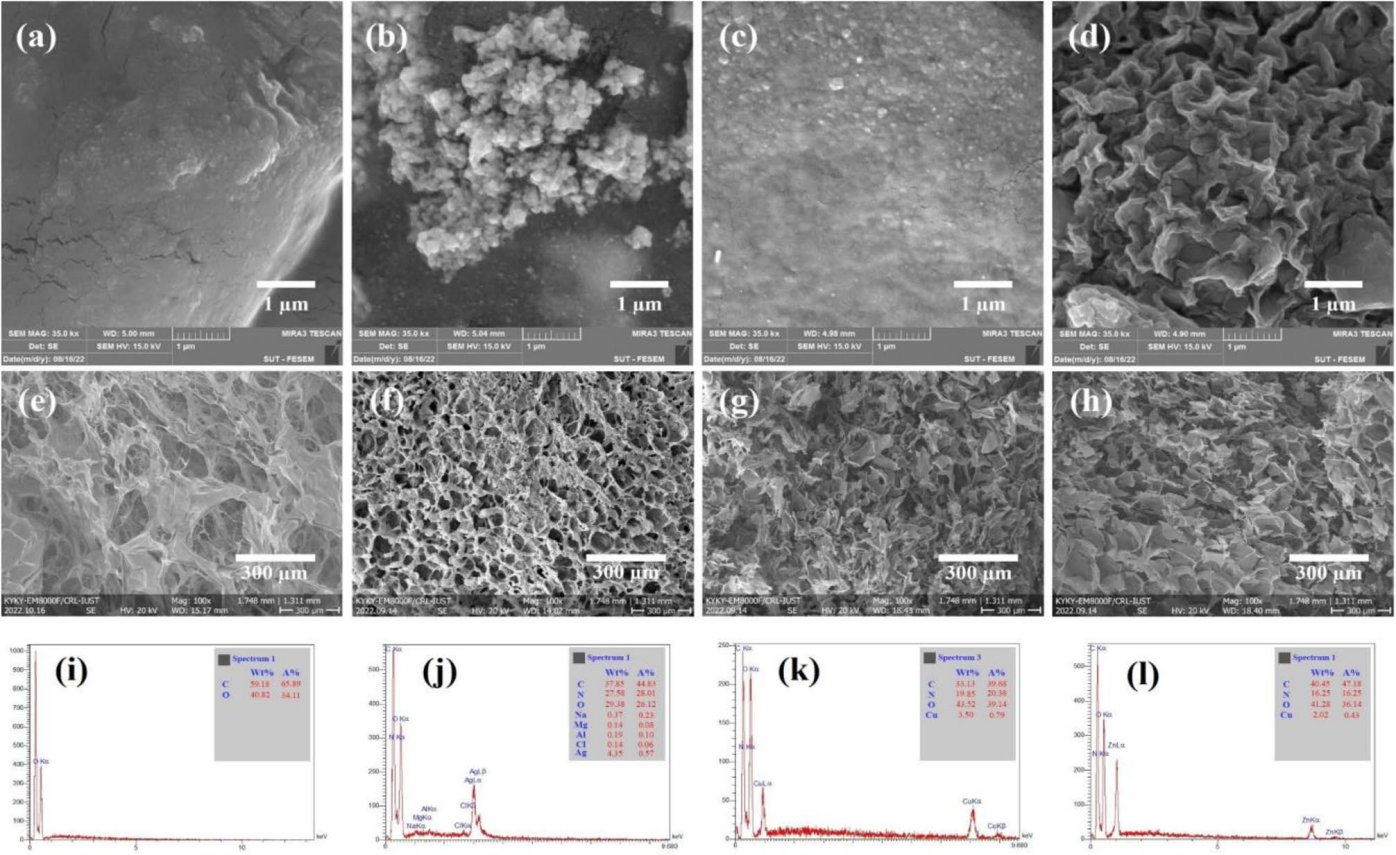
FESEM images of freeze-dried hydrogels of CS-g-GEL (**a**), CS-g-GEL-Ag^+^ (**b**), CS-g-GEL-Cu^2+^ (**c**) and CS-g-GEL-Zn^2+^ (**d**), the cross-sectional images of CS-g-GEL (**e**), CS-g-GEL-Ag^+^ (**f**), CS-g-GEL-Cu^2+^ (**g**) and CS-g-GEL-Zn^2+^ (**h**) freeze-dried hydrogels, and the EDX spectrum of CS-g-GEL (**i**), CS-g-GEL-Ag^+^ (**j**), CS-g-GEL-Cu^2+^ (**k**) and CS-g-GEL-Zn^2+^ (**l**) (wt% represents weight percentage and A% represents atomic percentage).

Since the hydrogels fabricated from CS-g-GEL-Metal ions have been designed for biomedical applications, including antibacterial activity, a thorough examination of their morphology and porous structure was required. Figure 5a–d shows FESEM images of freeze-dried hydrogels. In Fig. 5a, CS-g-GEL hydrogel displayed a dense structure after being freeze-dried. The density of the freeze-dried hydrogel can be influenced by various factors, such as the initial concentration of the gel precursor, the freezing rate, and the drying conditions. It was observed by FESEM images of freeze-dried hydrogels that CS-g-GEL-Metal ions hydrogels represent different structures in the presence of metal ions. Figure 5b shows the FESEM image of CS-g-GEL-Ag^+^ hydrogel as a snowflake-shaped structure. CS-g-GEL-Ag^+^ hydrogel was obtained as spherical, flocculated, aggregated particles. In addition, CS-g-GEL-Cu^2+^ hydrogel exhibited a dense structure after it was freeze-dried (Fig. 5c). Figure 5d illustrates the structures of the prepared CS-g-GEL-Zn^2+^ hydrogel sample. It was found that the hydrogel had a porous structure, and the pore size of the hydrogel was different. Figure 5e–h shows the cross-sectional images of CS-g-GEL and CS-g-GEL-Metal ions freeze-dried hydrogels. According to the results, interconnected microstructures were found in the CS-g-GEL and CS-g-GEL-Metal ions hydrogels. FESEM images showed porous structures formed by CS-g-GEL-Metal ions hydrogels, suggesting that these hydrogels have the ability to form porous networks. A study has reported the crosslinking of CS by the mentioned crosslinkers and the observation of porous structures formed by metal ions^35^. He et al.^48^ reported that graphene oxide/CS porous materials can facilitate the diffusion of metal ions. According to a study by Rogina et al., they found that with different metal ions and concentrations, the CS hydrogel structure can from a randomly porous structure. This indicates that the presence of metal ions has a significant impact on the morphology and organization of the CS hydrogel^36^. Figure 5i–l exhibits the EDX analyses of CS-g-GEL and CS-g-GEL-Metal ions hydrogels. The EDX spectrum of CS-g-GEL indicates the presence of C, N, and O (Fig. 5i), whereas the EDX spectrum of CS-g-GEL-Ag^+^, CS-g-GEL-Cu^2+^, and CS-g-GEL-Zn^2+^ (Fig. 5i–l) clearly shows the presence of the element silver, copper, and zinc, along with carbon, oxygen, and nitrogen, suggesting the successful formation of the metal-hydrogels. According to EDX results, CS-g-GEL-Ag^+^ showed the quantitative elemental composition of C (37.85 wt%), N (27.58 wt%), O (29.38 wt%), and Ag (4.35 wt%) with only a small amount of other elements impurities (Fig. 5j). As shown in Fig. 5k, the quantitative elemental composition of C (33.13 wt%), N (19.85 wt%), O (43.52 wt%), and Cu (3.50 wt%) were found for CS-g-GEL-Cu^2+^. Furthermore, CS-g-GEL-Zn^2+^ showed the quantitative elemental composition of C (40.45 wt%), N (16.25 wt%), O (41.28 wt%), and Zn (2.02 wt%) (Fig. 5l). When metal ions (Ag^+^, Cu^2+^, and Zn^2+^) are incorporated into the CS-g-GEL hydrogel, the EDX spectra demonstrate the presence of the respective metals alongside the existing carbon, nitrogen, and oxygen. This confirms the successful formation of metal-hydrogels, where the metal ions are incorporated within the hydrogel matrix.

### Antibacterial activity

#### Agar disk and well diffusion method

**Figure 6:**
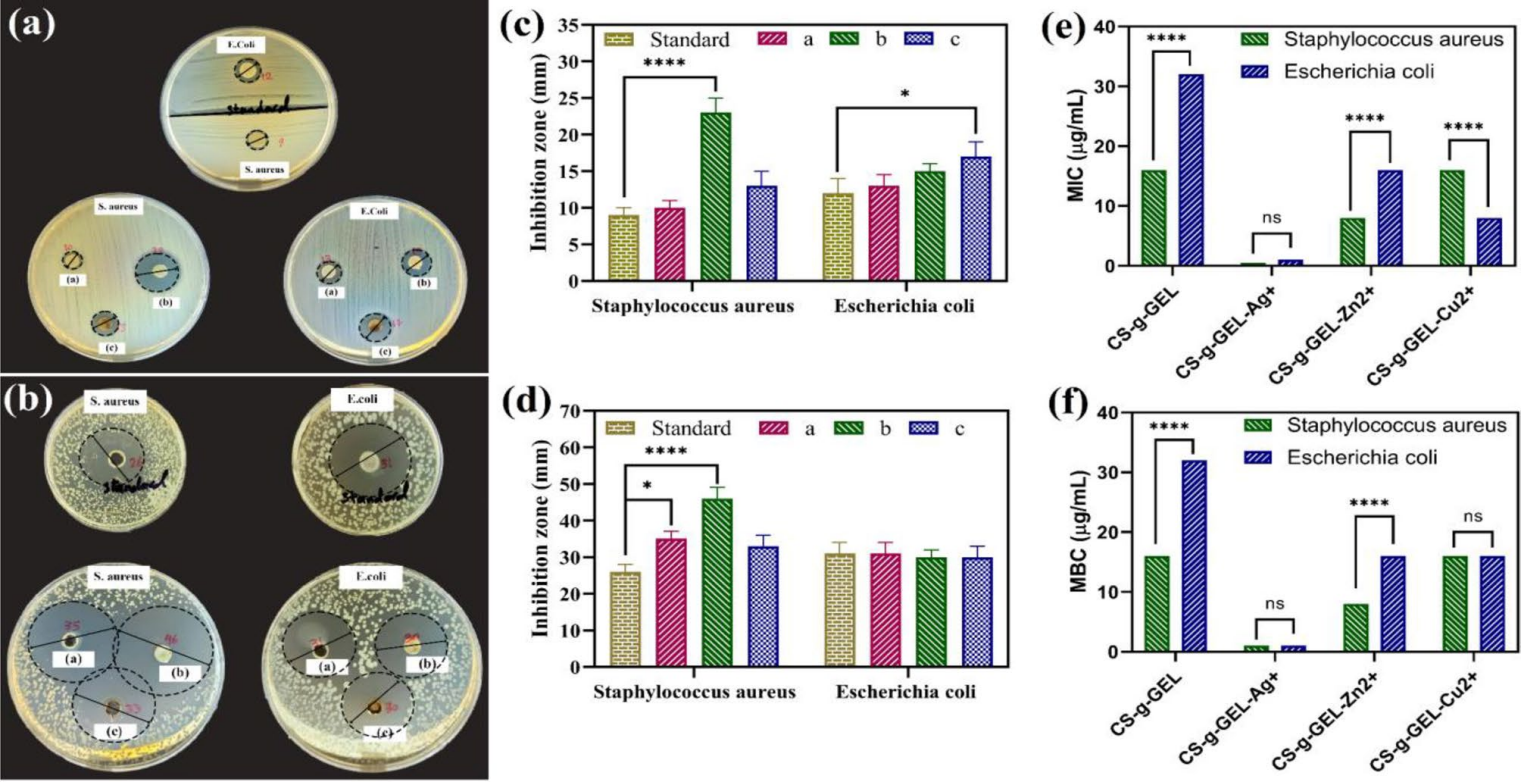
Antibacterial zone inhibition for CS-g-GEL (standard), CS-g-GE-Ag^+^ (**a**), CS-g-GEL-Zn^2+^ (**b**), and CS-g-GEL-Cu^2+^ (**c**) hydrogels against *Staphylococcus aureus* and *Escherichia coli* using (**a**, **c**) ADDM and (**b**, **d**) AWDM, and the MIC (**e**) and MBC (**f**) values of CS-g-GEL (standard), CS-g-GEL-Ag^+^, CS-g-GEL-Zn^2+^, and CS-g-GEL-Cu^2+^ against *Staphylococcus aureus* and *Escherichia coli* strains as determined by broth microdilution method (For all charts, ****p < 0.0001; ***p < 0.001; **p < 0.01; *p < 0.05).

AWDM and ADDM were used to investigate the antibacterial activity of CS-g-GEL (standard), CS-g-GEL-Ag^+^ (a), CS-g-GEL-Zn^2+^ (b), and CS-g-GEL-Cu^2+^ (c) hydrogels. Figure 6a, b illustrates the inhibition zones resulting from the screening test in ADDM, which indicates the antibacterial performance of prepared CS-g-GEL-Metal ions against *Staphylococcus aureus* (gram-positive) and *Escherichia coli* (gram-negative). It is worth noting that CS-g-GEL (standard) displayed excellent antibacterial activity against both *S. aureus* and *Escherichia coli*. In comparison with the other two kinds of hydrogels, CS-g-GEL-Zn^2+^ hydrogel displayed a higher antibacterial activity against *Staphylococcus aureus* (****p < 0.0001), while CS-g-GEL-Cu^2+^ demonstrated a relatively greater antibacterial activity against *Escherichia coli* (*p < 0.05). CS-g-GEL and zinc ions have weaker interactions with each other than silver and copper ions, which may explain the results. Furthermore, CS-g-GEL-Metal ions hydrogels showed a higher antibacterial capacity against *Staphylococcus aureus* and *Escherichia coli* when compared with CS-g-GEL, suggesting that both bacteria are susceptible to these hydrogels. The release of metal ions from the hydrogels may have contributed to the antibacterial properties of the material. Metal ions have a greater antibacterial effect when released at greater levels^49^. Figure 6d illustrates the inhibition zones observed following the screening test in AWDM, indicating the antibacterial properties of the CS-g-GEL and CS-g-GEL-Metal ions against *Staphylococcus aureus* and *Escherichia coli*. The inhibition zones observed in AWDM were much higher than the ADDM, which is expected due to the use of a larger number of samples, which leads to more diffusion of the substance in the solid medium. Similarly, to ADDM, in comparison with the other two kinds of hydrogels, CS-g-GEL-Zn^2+^ hydrogel displayed a higher antibacterial activity against *Staphylococcus aureus* (****p < 0.0001) (Fig. 6c). A higher inhibition zone was observed for CS-g-GEL-Ag^+^ hydrogel after CS-g-GEL-Zn^2+^ in AWDM than ADDM against *Staphylococcus aureus* (*p < 0.05). The activity of silver compounds has been attributed to several mechanisms, including binding to nucleic acids and proteins of bacteria, resulting in the loss of viability, resulting cellular damage, and inhibiting replication^50–52^. Silver compounds inactivate bacteria by interacting with biological molecules through ligand-exchange reactions^53^. Similarly, copper and zinc ions are observed to have similar effects^54^. However, Ag has a higher binding affinity to DNA molecules compared to Zn and Cu. DNA is essential for bacterial replication and survival. Ag ions can bind to the DNA molecule, disrupting its structure and inhibiting replication. This interference with DNA integrity further contributes to the antibacterial activity of Ag against *E. coli* and *Staphylococcus aureus*. CS-g-GEL and CS-g-GEL-Metal ions hydrogels exhibited similar inhibition zone and antibacterial activities against *Escherichia coli.* Antibacterial hydrogels have been shown to enhance wound healing by creating a moist environment around the wound site. By creating an optimal environment for these processes to occur, the prepared hydrogels can be a good candidate to help to speed up the healing process and reduce the risk of complications such as infection^55^.

#### The broth microdilution assay

The broth microdilution assay was used to determine the MIC and MBC values CS-g-GEL (standard), CS-g-GEL-Ag^+^, CS-g-GEL-Zn^2+^, and CS-g-GEL-Cu^2+^ at different concentrations (Fig. 6e, f). A MIC of 16 µg/mL was found for CS-g-GEL, indicating that concentrations below 16 µg/mL did not inhibit Staphylococcus aureus growth (Fig. 6e). In addition, the results showed that CS-g-GEL-Ag^+^ and CS-g-GEL-Zn^2+^ MIC values decreased by a significant amount in comparison to CS-g-GEL (standard), as observed MIC values of 0.5 µg/mL for CS-g-GEL-Ag^+^ and 8 µg/mL for CS-g-GEL-Zn^2+^ were within these ranges (Table 3). CS-g-GEL-Ag^+^ and CS-g-GEL-Zn^2+^ were found to have an enhanced bactericidal effect for *Staphylococcus aureus* when compared with CS-g-GEL and CS-g-GEL-Cu^2+^. In contrast to the MBC values observed for CS-g-GEL and CS-g-GEL-Cu^2+^ on *Staphylococcus aureus*, which was 16 µg/mL, MBC values of CS-g-GEL-Ag^+^ and CS-g-GEL-Zn^2+^ were significantly decreased to 1 µg/mL and 8 µg/mL, respectively (Fig. 6f). In addition, MIC and MBC values of prepared samples against *Escherichia coli* were determined. When compared to the MIC and MBC values of the standard sample against *Staphylococcus aureus*, CS-g-GEL (standard sample) and CS-g-GEL-Zn^2+^ had a reduced bactericidal effect against *Escherichia coli*. A MIC of 8 µg/mL was found for CS-g-GEL-Cu^2+^ on *Escherichia coli*, suggesting enhanced bactericidal activity compared to *Staphylococcus aureus*. In contrast, CS-g-GEL-Ag^+^ showed a MIC value of 1 µg/mL against *Escherichia coli, *suggesting reduced bactericidal activity. Ag ions have a strong affinity for sulfur-containing proteins present in bacterial cells. When Ag ions come into contact with the bacterial cell membrane, they can interact with proteins and enzymes involved in essential cellular processes. This disrupts the normal functioning of the cell and can lead to cell death. The interaction of Ag ions with sulfur-containing groups is more efficient compared to Zn and Cu ions, resulting in a higher antibacterial effect. The higher antibacterial activity of CS-g-GEL-Ag^+^ compared to CS-g-GEL-Zn^2+^, and CS-g-GEL-Cu^2+^ against *E. coli* and *Staphylococcus aureus* can be attributed to its strong affinity for sulfur-containing proteins, and higher binding affinity to DNA. These factors contribute to the disruption of essential cellular processes and DNA replication, ultimately leading to the effective killing of bacteria. Du et al. found that CS nanoparticles loaded with Ag^+^ were more effective against *E. coli* and *Staphylococcus aureus* than other metal ions^56^. The findings of this study align with other literature, demonstrating that CS-g-GEL-Ag^+^ exhibits higher antibacterial activity against E. coli and Staphylococcus aureus compared to CS-g-GEL-Zn^2+^ and CS-g-GEL-Cu^2+^.

**Table 3:** The MIC and MBC values of CS-g-GEL (standard), CS-g-GEL-Ag^+^, CS-g-GEL-Zn^2+^, and CS-g-GEL-Cu^2+^ against *Staphylococcus aureus* and *Escherichia coli* strains as determined by broth microdilution method.

Sample	*Staphylococcus aureus*		*Escherichia coli*	
	MIC (µg/mL)	MBC (µg/mL)	MIC (µg/mL)	MBC (µg/mL)
CS-g-GEL (standard)	16	16	32	32
CS-g-GEL-Ag^+^	0.5	1	1	1
CS-g-GEL-Zn^2+^	8	8	16	16
CS-g-GEL-Cu^2+^	16	16	8	16

#### Release study

**Figure 7:**
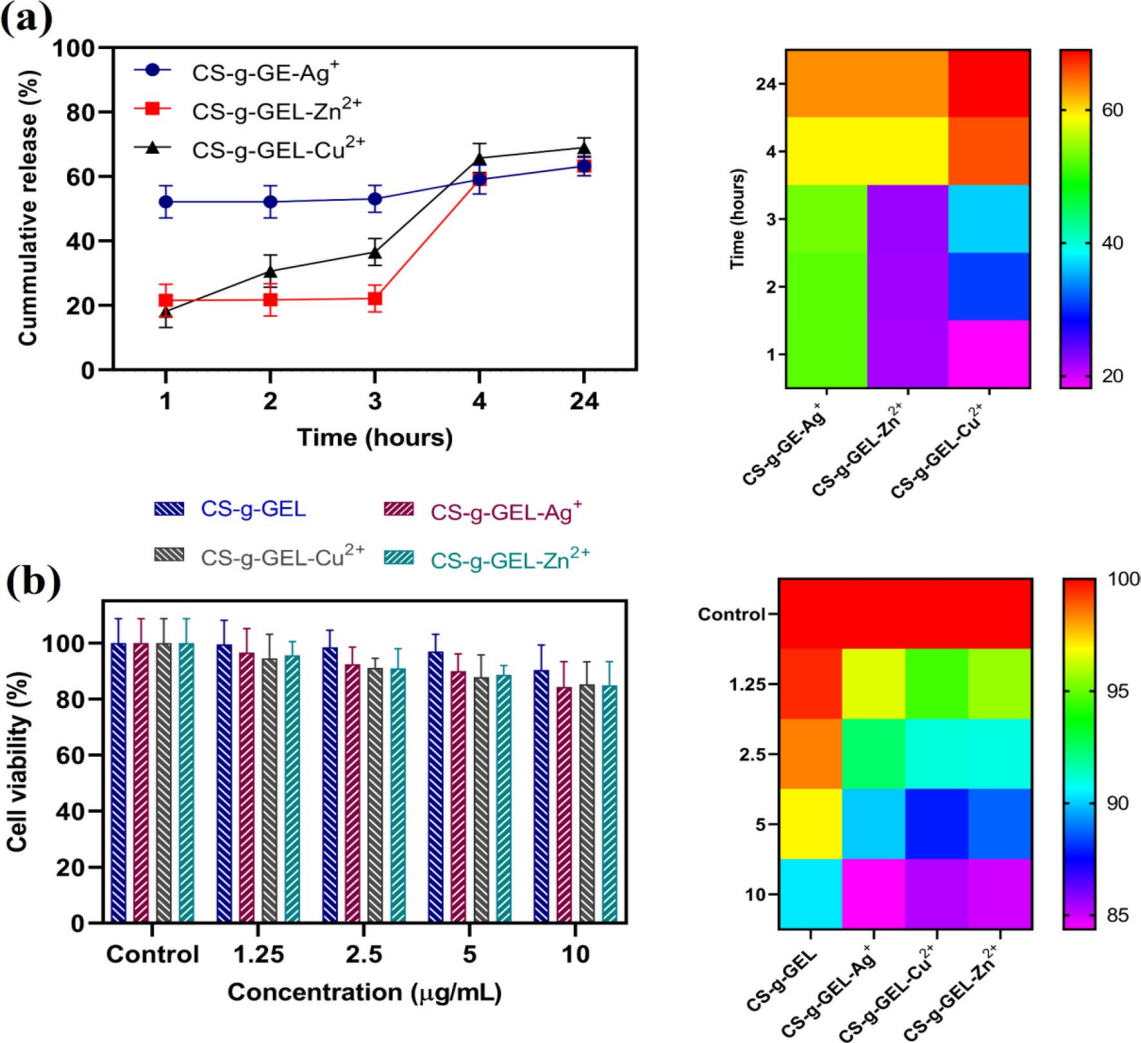
Release study of metal ions from CS-g-GEL, CS-g-GEL-Ag^+^, CS-g-GEL-Zn^2+^, and CS-g-GEL-Cu^2+^ hydrogels after 24 h at pH = 7.4 (**a**), and MTT assay of CS-g-GEL, CS-g-GEL-Ag^+^, CS-g-GEL-Zn^2+^, and CS-g-GEL-Cu^2+^ hydrogels against HFF-1 after 48 h (**b**).

Figure 7a shows the cumulative release percentages of Ag^+^, Zn^2+^, and Cu^2+^ ions from CS-based hydrogels at pH = 7.4. CS-g-GEL-Ag^+^ displayed a higher release percentage initially compared to CS-g-GEL-Zn^2+^ and CS-g-GEL-Cu^2+^. This suggests that there might be weak interaction between Ag^+^ ions and hydrogel matrix. A significant amount of metal ions has been released from the hydrogels by 24 h. The cumulative release exceeds 60% for all metals, with Ag^+^ having the highest release followed closely by Cu^2+^ and Zn^2+^. Thus, metal ion diffusion through the polymer network could be due to the swelling of CS-based hydrogels. The porous nature of the hydrogels likely permits water absorption, which could lead to expansion and a consequent loosening of the network, thus enabling the metal ions to move outward into the water. These differences in release profiles among metal ions may be attributed to variations in their affinity for chitosan chains. In addition, the release is also controlled by the strength of the ionic interactions within the hydrogel, when higher bonding between metal ions and hydrogel matrix may cause a slower release rate.

#### Cytotoxicity test

Cytotoxicity of CS-g-GEL, CS-g-GEL-Ag^+^, CS-g-GEL-Zn^2+^, and CS-g-GEL-Cu^2+^ was investigated for 48 h using the MTT assay. As shown in Fig. 7b, Human foreskin fibroblasts (HFF-1) cultured on CS-g-GEL (control sample) displayed a high cell viability percentage (> 90%). HFF-1 cells were chosen to study the cytotoxicity of these hydrogels because HFF-1 cells are a well-established and commonly used human fibroblast cell line. They are derived from normal foreskin tissue, making them a representative model for assessing the biocompatibility of biomaterials. However, CS-g-GEL-Ag^+^, CS-g-GEL-Zn^2+^, and CS-g-GEL-Cu^2+^ resulted in a small decrease in the viability of HFF-1 cells. Because of the release of metal ions, CS-g-GEL-Ag^+^, CS-g-GEL-Zn^2+^, and CS-g-GEL-Cu^2+^ complexes exhibited a limited decrease in viability toward HFF-1 cells. Based on these results, it can be concluded that metal ions did not have a significant cytotoxic effect. Similarly, Mutlu et al. reported that a small amount of cytotoxicity could be observed in MEF cells following preconditioning with chitosan-Cu complexes due to copper release^57^. A study by Nešović et al.^58^ found that MTT cytotoxicity tests confirmed biocompatibility of CS and poly(vinyl alcohol) with embedded Ag NPs hydrogels toward both MRC-5 and L929 fibroblast cell lines after 48 h. In another study, Nešović et al.^59^ found that MTT cytotoxicity tests on both MRC-5 and L929 cell lines showed low toxicity of all Ag/poly(vinyl alcohol)/chitosan/graphene hydrogel samples. Cheng et al.^60^ reported that cinnamaldehyde-modified crosslinked chitosan, and cinnamaldehyde-modified crosslinked chitosan-AgNPs hydrogels with the highest degree of crosslinking had cell viability of 90.8% and 80.2%, respectively, while the hydrogel with the lowest degree had cell viability of 48.4% and 37.6%, respectively. Hydrogels with low cytotoxicity have been found to be less likely to induce inflammation around the wound site. Inflammation can delay the healing process and increase the risk of infection, both of which can lead to further complications. By minimizing the risk of inflammation, the prepared hydrogels can promote a more efficient healing process and reduce the risk of complications, ultimately leading to better patient outcomes^61^. Table S1 represents a comparison between previous studies key characteristics in chitosan-based hydrogels incorporating ions.

## Conclusion

This study presented novel types of hydrogels with improved antibacterial activities that can be fabricated using the ionic complexation of CS with gelatin and metal ions (Ag^+^, Cu^2+^, and Zn^2+^). The gelation process is carried out without using an additional basic crosslinker and by mixing the CS, formaldehyde, and gelatin solutions with metal ions, making this process one of the novels reported procedures for the hydrogelation of materials. FTIR and XRD results showed the successful formation of complexes between CS-g-GEL and metal ions. FESEM images showed porous structures formed by CS-g-GEL-Metal ions hydrogels, suggesting that these hydrogels have the ability to form porous networks. The rheological properties of hydrogels indicated that their elastic nature as soft solids. When metal ions (Ag^+^, Cu^2+^, and Zn^2+^) are incorporated into the CS-g-GEL hydrogel, the EDX spectra demonstrate the presence of the respective metals alongside the existing carbon, nitrogen, and oxygen. Furthermore, the hydrogels demonstrated excellent antibacterial properties against *Staphylococcus aureus* and *Escherichia coli* bacteria. The broth microdilution method revealed lower MIC values for CS-g-GEL-Ag^+^, CS-g-GEL-Zn^2+^, and CS-g-GEL-Cu^2+^ against Staphylococcus aureus (0.5, 8, and 16) and Escherichia coli (1, 16, and 8). Similarly, the MBC values for these compounds were also lower against Staphylococcus aureus (1, 8, and 16) and Escherichia coli (1, 16, and 16). The MTT assay further confirmed the high biocompatibility of these compounds, showing 84.27%, 85.24%, and 84.96% cell viability at a concentration of 10 μg/ml for HFF-1 cells after 48 h. Therefore, these biocompatible hydrogels offer promising potential to be candidate for practical use as antibacterial materials in wound dressing applications in further researches and their wound healing properties can be evaluated.

## Materials and methods

### Materials

CS with medium molecular weight (MW 200,000 and 98% deacetylation) and gelatin was purchased from Sigma, USA. Silver nitrate, zinc acetate dihydrate, and copper (II) nitrate trihydrate were obtained from Merck (Darmstadt, Germany). Glacial acetic acid, sodium hydroxide (NaOH), aq. formaldehyde (37% in water), ammonia (25% in water), aq. hydrochloric acid 37%, ethanol (99.8%), and isopropanol (99%) were obtained from Neutron, Iran.

### Synthesis method

The hydrogels were synthesized by adapting and slightly modifying a methodology initially outlined by Farasati Far et al.^41^. To prepare CS-g-GEL-Ag^+^, CS-g-GEL-Zn^2+^, and CS-g-GEL-Cu^2+^ hydrogels, 1200 mg chitosan was dissolved using 1% aq. acetic acid (100 mL), and the solution was stirred for 8 h resulting a clear CS solution at ambient temperature. Then 2 mL of formaldehyde (37% in water) was added dropwise in 10 min. After 2 h, 500 mg of gelatin was added to the CS solution as a crosslinking agent and stirred for 48 h at 50 °C. In the next step, 200 mg of metal ions was added to 15 mL of the resulting mixture and stirred. The gelation time for each type of hydrogels is reported in Table 1. After the gelation was started, they were continued for 24 h to obtain CS-g-GEL-Ag^+^, CS-g-GEL-Cu^2+^, and CS-g-GEL-Zn^2+^ hydrogels. To fabricate the CS-g-GEL hydrogel, metal ions were not used. Instead, 20 mL of a 25% ammonia solution was added as a crosslinking agent during the novel process of gelation. Afterward, they were rinsed with ethanol and methanol to remove any unbonded and other unreacted material and were filtered via a vacuum pump and dried in oven (50 °C)^62,63^.

### Characterization of hydrogels

Avance Bruker DRX-500 spectrometer was used for the measurement of ^1^H NMR spectra. The solvent used was D_2_O, and the solvent signal acted as the basis for internal calibration (D_2_O: δ (1H) = 4.79 ppm(. In transmission mode, Fourier transform infrared (FTIR) spectra were measured using an Agilent Cary 630 FTIR spectrometer (Agilent Technologies/EUA). The superficial morphologies of CS-g-GEL, CS-g-GEL-Ag^+^, CS-g-GEL-Cu^2+^, and CS-g-GEL-Zn^2+^ were studied using Field-Emission Scanning Electron Microscopy (FESEM) (Topcon/Singapore). To coat the samples, a thin gold film of 16 nm was used. An energy-dispersive X-ray spectrophotometer (EDX) (JEOLJSM-600F) was employed for determining the composition of hydrogels, and then the CHNS analyzer was used to determine the percentage of elements in those samples (PerkinElmer, Series II). It was measured the X-ray diffraction spectra with an XRD 6000 diffractometer (Shimadzu, Kyoto, Japan) using a constant current of 30 mA at 40 kV and a step size of 0.05°. The rheological properties of the sample were evaluated using an Anton PaarMCR 502 rotational rheometer manufactured by Anton Paar GmbH in Graz, Austria. The standard concentric cylinder measuring geometry was employed, with a plate diameter of 25 mm and gap distance of 1 mm, along with a standard Peltier temperature device (CPTD200). A frequency sweep was conducted in the range of 1–50 Hz by applying a constant deformation of γ = 1 in the linear viscoelastic region while maintaining the temperature at 25 °C.

### Antibacterial assays

#### Agar disk diffusion method (ADDM)

The antibacterial activities of the prepared hydrogels against *Escherichia coli* (gram-negative) and *Staphylococcus aureus* (gram-positive) bacteria were assessed using the ADDM^64^. A fresh culture of the specified bacteria was prepared, and a bacterial suspension with a concentration of 0.5 McFarland (equivalent to 1.5 × 10^8^ CFU/mL) was created using a diluent solution (physiological serum 0.09%). The bacterial suspension was further diluted to achieve the desired cell count. Subsequently, the bacterial suspension was cultured on the surface of the plate using a sterile cotton swab. For ADDM, prepared/commercial sterile disks were utilized, and 15 µl of the samples were loaded onto the disk surface. The plates were incubated in an incubator for 48 h at a temperature of 30 ± 5 °C. Following incubation, the diameter of the growth inhibition halo was measured using a ruler or Vernier caliper and reported in millimeters. Each bacterial strain was analyzed three times, and mean values were calculated. The antibacterial activity of CS-g-GEL-Ag^+^ hydrogel was compared to CS-g-GEL hydrogel as a standard sample.

#### Agar well diffusion method (AWDM)

The AWDM was employed to evaluate the antibacterial activities of the hydrogels against *Escherichia coli* and *Staphylococcus aureus*^64^. Similar to the ADDM approach, a fresh bacterial culture and a bacterial suspension with a concentration of 0.5 McFarland were prepared. The bacterial suspension was appropriately diluted, and then cultured on the plate surface using a sterile cotton swab. In AWDM, wells with a diameter of 5 mm were created at suitable distances on the plate using a sterile cylinder. Using a sterile head sampler, 80 μL of the homogenized samples were inoculated into the wells. The plates were incubated in an incubator for 48 h at a temperature of 30 ± 5 °C. Following incubation, the diameter of the growth inhibition halo was measured using a ruler or Vernier caliper and reported in millimeters. Each bacterial strain was analyzed three times, and mean values were calculated. The antibacterial activity of CS-g-GEL-Zn^2+^ and CS-g-GEL-Cu^2+^ hydrogels was compared to CS-g-GEL hydrogel as a standard sample.

### Broth microdilution assay

A broth dilution method was used to determine the MIC^65^. The CS-g-GEL, CS-g-GEL-Ag^+^ (c), CS-g-GEL-Cu^2+^ (d), and CS-g-GEL-Zn^2+^ were gradually diluted in Muller–Hinton (MH) broth. There was an inoculation of bacteria to result in a concentration of 110^5^ CFU/ml. After 24 h of incubation at 37 °C, the minimum inhibitory concentration (MIC) was determined to be the concentration that had no visible growth in the tube. After 24 h of incubation at 37 °C, the MIC was determined to be the concentration that had no visible growth in the tube. As part of the evaluation of MBC, 100 µl of the sample was subsequently transferred from the tube that had no visible growth onto an MH agar plate. This was then incubated for another 24 h at 37 °C. As a result, the MBC was determined to be the concentration without bacterial growth in the tube. For each bacterium, the test was conducted in triplicate.

### In vitro release study

For this purpose, 20 mg of CS-g-GEL-Ag^+^ hydrogel was immersed in 30 ml of prepared PBS (pH = 7.4). 3 ml was taken from the release medium at predetermined intervals and replaced with fresh PBS. The released ions in aliquots were determined using UV–Vis spectrophotometry. The same method was performed for CS-g-GEL-Cu^2+^ and CS-g-GEL-Zn^2+^ hydrogels at pH = 7.4.

### Cell cytotoxicity test

In this study, Human foreskin fibroblasts (HFF-1) were cultured in a cell incubator, with DMEM (90%) containing FBS (10%) and penicillin/streptomycin (1%), with 5% CO_2_ at 37 °C. HFF-1 cells were collected with trypsin and resuspended in cold PBS. Then, viable cells were stained with 0.1% trypan blue and counted under the microscope before use in experiments. For the determination of cell viability, the MTT method was used. First, when the cells reached a state of encounter of approximately 80–90%, they were cultured into a 96-well plate with a density of 1 × 10^5^ cells/well for 24 h. After 24 h, the medium was removed, and the cells were washed with PBS, subsequently added samples containing DMEM medium at various concentrations, and incubated for an additional 24 h. The medium was then removed and replaced with the MTT assay, incubating the cells for 4 h. After 5 h, the MTT solution was removed, and we added 100 μl of DMSO. The plate was then analyzed by an ELISA microplate reader using 570 nm (ELx808i, BioTek. Instruments, USA), and cell viability percentage was estimated with the Eq. (1):

1 $${\%}\text{Viable cells} = \text{(Total number of viable cells per milliliter of aliquot/ Total number of cells per milliliter of aliquot)} \times 
100.$$

The cell viability of CS-g-GEL-Ag^+^, CS-g-GEL-Zn^2+^, and CS-g-GEL-Cu^2+^ hydrogels was compared to CS-g-GEL hydrogel as a control sample.

### Statistical analysis

Statistical analysis and curve fitting were performed using version 8 of GraphPad Prism software (GraphPad Software, Inc., San Diego, CA, USA). Data from three independent experiments were represented as means ± standard deviations. After confirming the normality and homoscedasticity of the dataset, one-way ANOVA showed statistical significance. For all analyses, statistical significance was pre-set at α = 0.05.

### Supplementary Information

Supplementary Information.

## Data Availability

The datasets generated during and/or analyzed during the current study are available from the corresponding author upon reasonable request.
