# Supplementary material for: Enhanced antibacterial activity of porous chitosan-based hydrogels crosslinked with gelatin and metal ions
Source: Sci Rep. 2024 Mar 29;14:7505. doi: 10.1038/s41598-024-58174-9 (PMC10980704; doi:10.1038/s41598-024-58174-9)
Supplement: Supplementary file 1 — Supplementary Information. [file 41598_2024_58174_MOESM1_ESM.docx]

**Supporting Information**

**Enhanced antibacterial activity of porous chitosan-based hydrogels crosslinked with gelatin and metal ions**

Bahareh Farasati Far^1^, Mohammad Reza Naimi-Jamal^1,*^, Mehdi Jahanbakhshi^2^, Alireza Hadizadeh^3^, Shiva Dehghan^4^, Shiva Hadizadeh^5^

^1^ Research Laboratory of Green Organic Synthesis and Polymers, Department of Chemistry, Iran University of Science and Technology, Tehran, Iran

^2^ School of Chemical Engineering, College of Engineering, University of Tehran, Tehran, Iran

^3^ Research Center for Advanced Technologies in Cardiovascular Medicine, Cardiovascular Diseases Research Institute, Tehran University of Medical Sciences, Tehran, Iran

^4^ School of Pharmacy, Shiraz University of Medical Sciences, Shiraz, Iran

^5^ Women Reproductive Health Research Center, Tabriz University of Medical Sciences, Tabriz, Iran

* Correspondence to: Mohammadreza Naimi-Jamal, Email: Naimi@iust.ac.ir, Tel: +982177451500, Fax: +982177491243, Postal Address: Iran University of Science and Technology, Narmak, Tehran, Iran.

**Table S1.** Comparison of previous studies key characteristics in chitosan-based hydrogels incorporating ions

| **Polymer** | **Ion** | **Mechanical Results** | **Antibacterial Results** | **Cytotoxity Results** | **Crosslinker** | **Limitations** | **Ref** |
| --- | --- | --- | --- | --- | --- | --- | --- |
| Pyrogallol-modified chitosan (GACS) - Polyvinyl alcohol (PVA) | - Zinc ions (Zn2+) were used as crosslinking and antibacterial agents | - PVA/GACS/Zn2+/LAE hydrogels have good mechanical properties. - The hydrogels have enhanced wound adhesion | PVA/GACS/Zn2+/LAE hydrogels efficiently kill bacteria. - PVA/GACS/Zn2+/LAE hydrogels accelerate wound healing | N/A | - Zinc ions (Zn2+) are used as the crosslinking agent | - Lack of Cytotoxicity Assessment  - Incomplete Biomedical Suitability Assessment: Without cytotoxicity data, the overall suitability of the hydrogels for biomedical applications, particularly in contact with living tissues, remains unclear | ^54^ |
| CS  - Beta-glycerophosphate (βGP) | - Copper ions (Cu2+) were used in the study. - Diethyldithiocarbamate (DDC−) and copper ions (Cu2+) were used in combination | - CS-bGP system is closer to a liquid than a solid at ambient temperature. - The gel has thermosensitive properties and forms a gel at body temperature | - Lipogel killed 98.7% of MRSA biofilms. - Lipogel killed 99.9% of S. epidermidis biofilms | - CS-bGP showed negligible cytotoxic effects on fibroblast cells. - Components released from CS-bGP showed no toxicity over 72 hours | - The crosslinker used in the study is diethyldithiocarbamate (DDC-). - The crosslinker forms a complex with copper ions (Cu2+) | Physical structure of liposomes can alter during lyophilization process - Drug leakage can occur due to rupture of lipid bilayer or liposome aggregation/fusion during drying process | ^55^ |
| Carboxymethyl CS  - Polyvinyl alcohol | - Zinc acetate (ZnAc2) is used as a catalyst. - Zinc ions are adsorbed onto the composite hydrogel | - CMCh/PVA composite hydrogel has a compressive strength of 1.96 MPa. - Dried composite sample has a water absorption capacity of 1860% | - The composite hydrogel exhibits improved antibacterial activity. - The antibacterial activities against Escherichia coli and Staphylococcus aureus were studied | N/A | - Poly(ethylene glycol) diglycidyl ether (PEGDE) is the crosslinker | - No information provided on in vivo efficacy and safety  - No mention of potential side effects or limitations | ^56^ |
| Poly(vinyl alcohol)/chitosan (PVA/CS) hydrogel. | - Magnesium ions (Mg ions) are used in the hydrogel | N/A | - PVA/CS-MgO-BPNS hydrogel kills more than 99.9% of Staphylococcus aureus and Escherichia coli | N/A | N/A | Lack of Comprehensive Cytotoxicity Assessment: the absence of detailed cytotoxicity data represents a limitation in fully understanding the potential impact on cells | ^57^ |
| Carboxymethyl CS is used as the polymer. | - Ca2+ ion is used for crosslinking the hydrogel | - High stretchability, toughness, and tissue adhesion  - Covalent and ionic crosslinking for enhanced mechanical properties | - The hydrogel dressing has enhanced antibacterial properties.  - The efficacy of the hydrogel in accelerating bacterial-infected wound healing is demonstrated | - Hydrogel exhibits excellent cytocompatibility  - No information provided about cytotoxicity results | - Silane - Ca2+ | Incomplete Antibacterial Assessment: the lack of specific antibacterial results or details on the antimicrobial mechanisms hinders a comprehensive evaluation of its antibacterial performance.  Limited Crosslinker Information: The study provides information about the use of silane and Ca2+ as crosslinkers. However, there is a lack of detail regarding the specific roles and interactions of these crosslinkers in the hydrogel network, limiting a thorough understanding of the crosslinking mechanisms | ^58^ |
| Chitosan  - Carboxymethyl CS (CMCTS) | Silver ions (Ag+) are used in the preparation | - Composite hydrogel has good mechanical strength. - Hydrogel exhibits self-healing ability | - The composite hydrogel exhibits excellent antibacterial activities.  - The antibacterial activities are effective against both S. aureus and P. aeruginosa | - CTS/CMCTS/AgNPs hydrogel showed high biocompatibility |  | - Lack of Specific Mechanical Property Quantification: there is a limitation in the absence of specific quantification or detailed characterization of these mechanical properties. Providing quantitative measures would enhance the precision of the mechanical assessment.  - Limited Antibacterial Specifics: there is a limitation in the lack of specific details regarding the antibacterial mechanisms or quantitative data. A more detailed antibacterial analysis would provide a more comprehensive understanding of the hydrogel's effectiveness.  - Incomplete Cytotoxicity Information | ^59^ |
| Carboxymethyl chitosan (CMCTS)  Polyacrylic acid (PAA) | - Aluminum (III) ions (Al3+)  - Metal ions (Fe(III)) | - Tensile fracture stress increased from 83.1 kPa to 190.9 kPa.  - Tensile fracture strain increased from 1673 to 1930 | - Log reduction of S.aureus: 3.0 to 3.8  - Log reduction of E.coli: 3.8 to 4.5 | - RGR of positive control decreased to 48.9.  - Cells cultivated well in experiment groups | - Aluminum (III) ions (Al3+) - Carboxymethyl chitosan nanoparticles (CMCS NPs) | - Low mechanical property and limited self-healing ability  - Potential cytotoxicity and poor recyclability | ^60^ |
| Poly(vinyl alcohol) (PVA) - CS (CHI) | - Silver ions (Ag+)  - Potassium ions (K+) | - High gelation degrees of 94.0 and 95.6  - Good mechanical properties of the hydrogels | - Susceptibility to bacterial infection  - Poor regulation of moisture | - All hydrogels were non-toxic or mildly cytotoxic. - 0.25AgPVA0.1CHI and 0.25AgPVA0.5CHI hydrogels showed significant decrease in viability | N/A | - Limited Antimicrobial Efficacy: the limitation lies in their susceptibility to bacterial infection, highlighting a potential drawback in their effectiveness as antibacterial materials.  - Cytotoxicity Concerns: While the majority of the hydrogels were deemed non-toxic or mildly cytotoxic, the 0.25AgPVA0.1CHI and 0.25AgPVA0.5CHI hydrogels showed a significant decrease in viability. This raises concerns about the cytotoxicity of specific formulations, indicating a need for further investigation and optimization to ensure their safety for potential biomedical use | ^61^ |
| Carboxymethyl CS (CMCh) - Chitosan | Zinc ions were used in the study | N/A | - Antibacterial activity of CMCh/ZnO hydrogel against Escherichia coli and Staphylococcus aureus. - S. aureus is more susceptible to nanocomposite hydrogel compared to E.coli | N/A | - The crosslinker used in the study is zinc nitrate solution | - Limited information on the antimicrobial mechanism of ZnO nanoparticles. - Lack of information on the long-term stability of the nanocomposite hydrogels | ^62^ |
| Carboxymethyl CS (CMCh) | - Copper (II) chloride (CuCl2) solution - Sodium hydroxide (NaOH) | N/A | - Bacterial population reduced by 50% after 1 hour incubation - No viable bacterial colonies found after 4 hours incubation | N/A | - The crosslinker used in this study is copper (II) chloride (CuCl2) solution.  - Sodium hydroxide is used as a base for the oxidation of copper ions | - Lack of Mechanical Property Analysis: The absence of mechanical results limits a comprehensive understanding of the hydrogel's suitability for specific applications.  - Limited Information on Cytotoxicity: the lack of cytotoxicity results presents a limitation in fully assessing the biocompatibility and safety of the CMCh hydrogel.  - Incomplete Crosslinking Mechanism Explanation: the detailed mechanism of the crosslinking process is not explained | ^63^ |
| β-cyclodextrin-modified chitosan (CS-CD) | - Supramolecular hydrogel  AgNO3 | N/A | - The supramolecular hydrogel exhibited a high antibacterial effect.  - No specific numeric result is provided in the abstract | N/A | - The crosslinker is β-cyclodextrin-modified chitosan (CS-CD).  - The crosslinker is AgNO3 | - Lack of Mechanical Property Analysis: The absence of mechanical results limits a comprehensive understanding of the hydrogel's mechanical stability and applicability in various settings.  - Limited Information on Cytotoxicity: the lack of cytotoxicity results represents a limitation in fully assessing the biocompatibility and safety of the CS-CD hydrogel. Cytotoxicity data is crucial for evaluating the potential use of the hydrogel in biomedical applications.  - Incomplete Crosslinking Mechanism Explanation: The use of β-cyclodextrin-modified chitosan (CS-CD) and AgNO3 as crosslinkers is mentioned, but there is a lack of detailed information on the crosslinking mechanism | ^64^ |
| Carboxymethyl CS (CMCh) used as the polymer. | - Ag+, Cu2+, and Zn2+ ions were used. | - Hydrogels exhibited remarkable moldability to form free standing objects. | - The supramolecular hydrogels showed excellent antibacterial activity.  - The specific numeric result is not provided | N/A | - Metal ions (Ag+, Cu2+ and Zn2+)  - Carboxylic, amino and hydroxyl groups of CMCh chains | - Limited Mechanical Property Assessment:  The study lacks quantitative analysis of mechanical properties such as tensile strength, elasticity, or compressive strength, preventing a comprehensive understanding of the hydrogel's mechanical behavior.  - Unaddressed Cytotoxicity Concerns:  - The study does not provide data or information on cytotoxicity results, leaving a significant gap in the evaluation of the hydrogel's safety for use in biomedical applications.  - Incomplete Discussion on Biomedical Applications:  - This study does not provide a detailed discussion on specific biomedical applications or potential challenges in translating the findings into practical solutions. | ^33^ |
| Chitosan | - Ag+, Cu2+, and Zn2+ | The elastic nature of the CS-g-GEL-Metal ions hydrogels is likely due to the crosslinked network structure of the hydrogel, which allows it to resist deformation and maintain its shape | MIC for CS-g-GEL-Ag+, CS-g-GEL-Zn2+, CS-g-GEL-Cu2+ (0.5, 8, 16) µg/mL in Staphylococcus aureus and (1, 16, 8) µg/mL in Escherichia coli | Human foreskin fibroblasts (HFF-1) cultured on CS-g-GEL (control sample) displayed a high cell viability percentage (>90%). Although, CS-g-GEL-Ag+, CS-g-GEL-Zn2+, and CS-g-GEL-Cu2+ resulted in a small decrease in the viability of HFF-1 cells, all of the viabilities were over 80%% | Gelatin, - Metal ions (Ag+, Cu2+ and Zn2+) | Lack of In vivo studies | This work |
